# The ACTyourCHANGE in Teens Study Protocol: An Acceptance and Commitment Therapy-Based Intervention for Adolescents with Obesity: A Randomized Controlled Trial

**DOI:** 10.3390/ijerph18126225

**Published:** 2021-06-09

**Authors:** Anna Guerrini Usubini, Roberto Cattivelli, Vanessa Bertuzzi, Giorgia Varallo, Alessandro Alberto Rossi, Clarissa Volpi, Michela Bottacchi, Sofia Tamini, Alessandra De Col, Giada Pietrabissa, Stefania Mannarini, Gianluca Castelnuovo, Enrico Molinari, Alessandro Sartorio

**Affiliations:** 1 Istituto Auxologico Italiano IRCCS, Psychology Research Laboratory, 20145 Milan, Italy; r.cattivelli@auxologico.it (R.C.); g.varallo@auxologico.it (G.V.); c.volpi@auxologico.it (C.V.); m.bottacchi@auxologico.it (M.B.); g.pietrabissa@auxologico.it (G.P.); gianluca.castelnuovo@auxologico.it (G.C.); molinari@auxologico.it (E.M.); 2 Department of Psychology, Catholic University of Milan, 20123 Milan, Italy; vanessa.bertuzzi@unicatt.it; 3 Department of Philosophy, Sociology, Education, and Applied Psychology, Section of Applied Psychology, University of Padova, 35139 Padova, Italy; a.rossi@unipd.it (A.A.R.); stefania.mannarini@unipd.it (S.M.); 4 Interdepartmental Center for Family Research, University of Padova, 35139 Padova, Italy; 5 Istituto Auxologico Italiano, IRCCS, Experimental Laboratory for Auxo-Endocrinological Research, 28824 Piancavallo (VB), Italy; s.tamini@auxologico.it (S.T.); a.decol@auxologico.it (A.D.C.); sartorio@auxologico.it (A.S.); 6 Istituto Auxologico Italiano, IRCCS, Division of Auxology, 28824 Piancavallo (VB), Italy

**Keywords:** obesity rehabilitation, adolescents, acceptance and commitment therapy, psychological well-being, psychological flexibility

## Abstract

This Randomized Controlled Trial [(RCT) aims to evaluate the effectiveness of a brief Acceptance and Commitment Therapy (ACT)-based intervention combined with treatment as usual (TAU) compared to TAU only in improving psychological conditions in a sample of adolescents with obesity (body mass index, BMI > 97th percentile for age and sex) within the context of a wider multidisciplinary rehabilitation program for weight loss. Fifty consecutive adolescents (12–17 years) of both genders with obesity will be recruited among the patients hospitalized in a clinical center for obesity rehabilitation and randomly allocated into two experimental conditions: ACT + TAU vs. TAU only. Both groups will attend a three-week in-hospital multidisciplinary rehabilitation program for weight loss. The ACT + TAU condition comprises a psychological intervention based on ACT combined with a standard psychological assessment and support to the hospitalization. The TAU comprises the standard psychological assessment and support to the hospitalization. At pre- to post-psychological intervention, participants will complete the Avoidance and Fusion Questionnaire for Youth, the Psychological Well-Being Scale, the Depression Anxiety Stress Scale, the Difficulties in Emotion Regulation Scale, and the Emotional Eating subscale of the Dutch Eating Behavior Questionnaire to assess psychological well-being as the primary outcome and experiential avoidance, psychological distress, emotional dysregulation, and emotional eating as secondary outcomes. Repeated-measures ANOVAs (2 × 2) will be conducted. The study will assess the effectiveness of a brief ACT-based intervention for adolescents with obesity in improving their psychological conditions by targeting specific core processes of the ACT framework (openness, awareness, and engagement). Future directions of the study will assess whether these psychological processes will contribute to addressing long-term weight loss.

## 1. Introduction

Obesity in childhood and adolescence is becoming a major public health concern. In this respect, recent estimates pointed out that globally, 124 million children and adolescents, aged between 5 and 19 years, were obese [1] In Europe, the prevalence of overweight and obese children and adolescents aged between 5 and 19 years is reported around 19%, with a higher prevalence in southern European countries [2].

Obesity in children and adolescents is associated with a higher risk of developing several physical and psychological problems that have an impact on their emotional development [3]. Research has reported associations between childhood and adolescent obesity and some form of psychopathology, such as depression [4], anxiety, and attention-deficit/hyperactivity disorder [5]. Children and adolescents with obesity are more likely to suffer from psychosocial disadvantages such as discrimination, social isolation, and bullying because of their weight [6,7,8,9]. Moreover, obesity in childhood and adolescence is associated with poor body image, low self-esteem [10], and low quality of life and well-being [11]. 

This evidence suggests that obesity is a complex disease that may dramatically impair psychological health, representing a risk factor for children and adolescents’ future development. This aspect calls for a better understanding of the associated risk factors and requires a multidisciplinary approach—entailing medical, nutritional, physical, and psychological components—aimed to address not only weight loss but also improving psychological conditions and treating obesity-related comorbidities [12,13]. 

The development of dysfunctional eating habits is one of the main factors linked to body weight, and it can be considered a barrier to weight loss [14,15]. Evidence from the literature suggests that unhealthy and dysfunctional eating habits could be related to a failure in emotional regulation strategies that leads individuals to eat in response to difficult emotions, as in the case of emotional eating defined as the tendency to eat in response to negative feelings [16]. 

Emotional regulation is defined as the ability to manage emotions, which means to recognize, understand, accept, and modulate flexible responses to emotions [17]. High emotion regulation allows individuals to act accordingly with personal goals, even in the presence of difficult emotions, while controlling for impulsive behaviors. On the contrary, when emotional regulation is lacking, emotional eating is undertaken to regulate difficult feelings. 

Since emotional eating was related to higher consumption of sweet and fatty food, as an attempt to regulate negative feelings, it was found to be positively associated with weight gain and obesity, both in adults [18] and in younger populations [19]. 

A theoretical model [20] for explaining disordered eating suggests that momentary stimuli such as the availability of tasty food in the environment, in combination with a modern sedentary lifestyle, accompanied by difficult emotions, can lead people to move toward behaviors perceived to increase or maintain a hedonic state of pleasure, such as eating tasty food immediately. The model also suggests that values clarity and behavioral commitment [to connect healthy eating habits to important life values], awareness [of their automatic thoughts], and distress tolerance [negative internal states] are specific self-regulation skills that can help individuals to deal with difficult emotions and control for their behaviors. Such skills are the main focus of Acceptance and Commitment Therapy. 

Acceptance and Commitment Therapy (ACT) [21] is a third-wave CBT raised in the last twenty years. ACT aims to promote psychological flexibility, which is defined as the ability to live in the “here and now” current moment consciously while implementing behaviors to live in line with one’s values. This can be seen in opposition to experiential avoidance (or psychological inflexibility), which is the attempt to avoid unpleasant internal states, such as thoughts, feelings, and bodily sensations. Psychological flexibility is an important component for health promotion [22], since it was found that it helps to commit to behaviors that are coherent with deeply held values and a life directions that a person chooses as important and meaningful in his life [23]. Concurrently, it entails staying in contact with the present moment, regardless of unpleasant internal states, such as thoughts and emotions, which are considered as a normal part of human life. By shifting the focus from efforts to change dysfunctional thoughts and feelings, in ACT, the emphasis is placed on promoting behaviors that are consistent with personal values and goals even when dysfunctional thoughts and feeling are present, by promoting acceptance and awareness of personal internal states.The promotion of psychological flexibility is based on three pillars: openness, awareness, and engagement. Openness refers to the willingness to develop an open and acceptable attitude toward one’s personal internal states such as thoughts, emotions, and bodily sensations; awareness refers to the ability to act intentionally with awareness about personal thoughts and sensations, without automatically reacting; engagement refers to engage oneself in committed behaviors related to personal values—that is, chosen life directions [24].

Several ACT-based protocols have been successfully applied to adolescents targeting a broad range of conditions, such as cystic fibrosis [25], chronic pain [26], ADHD [27], and at-risk adolescents [28]. In a pilot study conducted by Hayes, Boyd, and Sewell [29] thirty adolescents referred to a psychiatric outpatient service were randomly assigned to two conditions: in one, they received an ACT intervention; in the other, participants received a standard intervention for depression. The results showed a significant decrease in depressive symptoms in patients undergoing the ACT intervention. In a study conducted by Kemani and colleagues [30], an ACT-based intervention was applied to adolescents with chronic pain and their family members. Results showed improved acceptance of chronic pain and psychological flexibility in adolescents as well as decreased depressive symptoms in their parents. ACT has also been applied to adolescents with post-traumatic stress disorder [31], proving a reduction of symptoms after a 10-week intervention. 

Unfortunately, there are not many studies in the literature concerning the application of an ACT protocol for adolescents with overweight/obesity problems. However, evidence suggests that psychological flexibility is an important key factor of psychological health, since it allows people to engage in meaningful challenges, following a person’s self-concept and important life domains [22]. To the best of our knowledge, only one pilot study published in 2019 [32], evaluating the effectiveness of an ACT-based psychological intervention applied in combination with a 16-week lifestyle behavioral modification program in a sample of obese adolescents, was found. This study showed that the intervention was effective in promoting a reduction in BMI and an overall improvement in physical activity and psychological status of obese adolescents. Furthermore, the intervention was positively evaluated by both the adolescents and their parents who participated in the study.

Given these premises, the present study aims to evaluate the effectiveness of a brief ACT-based psychological intervention, compared to TAU in improving psychological conditions in a sample of adolescents with obesity attending a multidisciplinary rehabilitation program for weight loss. 

## 2. Materials and Methods

### 2.1. Study Design 

A superiority Randomized Controlled Trial (RCT) with parallel groups will be conducted with an ACT-based intervention plus TAU compared with TAU only for adolescents with obesity attending a multidisciplinary rehabilitation program for weight loss. 

### 2.2. Participants

Participants will be consecutively recruited at the admission to the division of Auxology, Istituto Auxologico Italiano IRCCS, Piancavallo (VB), which is located in the North-West of Italy, a specialized clinical center [i.e., third level] for weight loss and obesity rehabilitation.

Participants of both genders will be eligible for the study if they meet the following criteria: (1) aged between 12 and 17 according to the classification of the pubertal status [33]; (2) BMI > 97th centile according to age- and sex-specific Italian charts [34]; (3) Italian mother tongue; (4) written and informed consent to participate from both parents and written assent from participants. Patients will be excluded if they have any physical or psychiatric problems according to the Diagnostic and Statistical Manual of Mental Disorders (DSM-5) criteria that could compromise participation in the study. 

### 2.3. Measures

All demographical (gender, age, nationality, educational level, and family composition) and clinical data will be collected via self-report using Italian validated and widely used questionnaires. The following psychological measures will be collected pre (Time 0/week 1) and post (Time 1/week 3) psychological intervention. 

#### 2.3.1. Primary Outcomes 

Psychological Well-Being. The Psychological Well-Being Scales (PWB) [35] will be administered to assess psychological well-being. It is a self-report measure that explores six dimensions: self-acceptance, positive relationships with others, autonomy, environmental control, personal growth, and life purpose. The questionnaire consists of 18 items rated on a 4-point Likert scale ranging from 1 (“completely disagree”) to 4 (“completely agree”). The Italian version [36], assessed for Italian adolescents [37], is reported to have good psychometric properties (test–retest correlation coefficients: Self-acceptance: *r* = 0.82; Positive relationships: *r* = 0.81; Autonomy: *r* = 0.21; Environmental control: *r* = 0.31; Life purpose: *r* = 0.81; Personal growth: *r* = 0.78).

#### 2.3.2. Secondary Outcomes 

Experiential avoidance and fusion. The Avoidance and Fusion Questionnaire for Youth (AFQ-Y) [38] will be administered as a measure of experiential avoidance and fusion in adolescents. It consists of 8 items rated on a 5-point Likert scale ranging from 0 [“not at all true”] to 4 [“absolutely true”]. The Italian version [39] is reported to have moderate internal consistency (Cronbach’s alpha = 0.69) and good test–retest reliability (*r* = 0.64). 

Psychological distress. The *Depression Anxiety Stress Scale* (DASS-21) [40] will be administered as a measure of psychological distress, which is widely used in samples of adolescents [41,42]. It is a self-report instrument that measures several negative internal states: depression, anxiety, and stress. It consists of 21 items rated on a 4-point Likert scale, ranging from 0 to 3. The Italian version [43] showed good psychometric properties (Cronbach’s alpha values of subscales ranged from 0.83 to 0.91. The Cronbach’s alpha of the total score was = 0.92).

Emotional dysregulation. The Difficulties in Emotion Regulation Scale (DERS) [17] will be administered to assess difficulties in emotional dysregulation. This is a self-report questionnaire consisting of 36 items rated on a 5-point Likert scale ranging from 1 (“almost never”) to 5 (“almost always”), which explores the following subscales: non-acceptance of negative emotions, inability to undertake purposeful behavior when experiencing negative emotions, difficulty in controlling impulsive behavior when experiencing negative emotions, limited access to emotion regulation strategies that are considered effective, lack of awareness of one’s emotions, and lack of understanding of the nature of one’s emotional responses. The Italian version [44], which is widely used in samples of adolescents [45,46], is reported to have good psychometric properties (Cronbach’s alpha values ranged from 0.77 to 0.89. The Cronbach’s alpha of the total score was 0.92).

Emotional eating. The Emotional Eating subscale of the Dutch Eating Behavior Questionnaire (DEBQ) [47] will be administered to assess emotional eating. The DEBQ is a self-report questionnaire used to detect eating behaviors. The Emotional Eating subscale consists of 13 items, rated on a 5-step Likert scale ranging from 0 (“never”) to 4 (“almost always”). The Italian version [48] showed good psychometric properties (Cronbach’s alpha = 0.97). The DEBQ was chosen as a measure of emotional eating in our sample, since no other validated instruments are available, except for the DEBQ version for parents [49], which is unfortunately unsuitable for parents of our sample of hospitalized adolescents. 

### 2.4. Randomization Procedure

Participants will be randomly assigned to the experimental (ACT+ TAU) or control (TAU) group. We will perform simple randomization with a 1:1 allocation ratio using the web site Randomization.com (http://www.randomization.com) (accessed on 8 June 2021). Randomization will occur after the baseline measurements in order to ensure similar baseline characteristics of groups.

Allocation concealment will be ensured, since all the patients will generate an anonymous code that will be associated with the randomization generated by the program. Researchers will be blind to the association made.

### 2.5. Procedures 

The trial will be conducted within the context of a three-week in-hospital multidisciplinary rehabilitation program for weight loss based on nutritional, physical, and psychological rehabilitation [50,51,52], according to the Italian National Health System recommendations. As part of the nutritional program, adolescents will be assessed by a staff dietician and placed on an individualized hypocaloric diet composed of 53% carbohydrates, 26% fat, and 21% protein [53] and a fluid intake of at least 1500 mL per day^−1^. They will also follow a daily nutritional counseling program provided both in individual and group settings, comprising dietetics lessons. As for physical rehabilitation, young patients will participate in a physical activity program consisting of five group training sessions per week lasting about 45–60 min per day of physical therapy comprising aerobic activities such as walking and cycling under the supervision of a therapist. The physical program also entails 2 h per week of aerobic leisure activities. As far as the psychological component is concerned, patients will take part in psychological counseling sessions that are provided at least once a week lasting about one hour each, carried out by a clinical psychologist, and aimed at promoting a healthy lifestyle and addressing psychological factors related to the onset of dysfunctional lifestyle habits. 

At week 1 of the hospitalization, all the patients will be screened for admission to the study with a clinical interview conducted by a clinical psychologist blinded to research aims to provide information about the study and assess the eligibility criteria according to the Diagnostic and Statistical Manual of Mental Disorders (DSM-5). Weight and height, to calculate BMI, will be measured by the internal medical team. Once we obtain informed consent to participate from parents and written assent from participants, young patients recruited for the study will be randomly assigned into two conditions. The experimental group will attend TAU plus a brief ACT-based intervention that comprises three sessions, provided once a week, lasting about one hour each, while the control group will attend TAU only. 

At pre (Time 0/week 1) and post-psychological intervention (Time 1/week 3), patients of both conditions will be invited to fill the questionnaires under the supervision of a member of the research team. 

In case of any form of psychological discomfort due to the participation in the intervention or any doubt or need for information concerning the trial, participants will have the opportunity to consult the psychologist responsible for the study. Once enrolled, patients can withdraw from the study at any time. This will not affect their future treatment. 

The clinical psychologist who will conduct the sessions, participants, and observers will be blinded to research aims. 

Completed questionnaires will be stored in a room used for research in the hospital, which is accessible only for research team members. Data will be stored on password-protected files kept for five years after the end of the trial. 

The study protocol was approved by the Ethical Committee of the Istituto Auxologico Italiano (registration number 2021_01_26_03) and registered on ClinicalTrials.gov (last update on 7 June 2021). All procedures will be conducted following the Helsinki Declaration and its later advancements.

**Figure 1:**
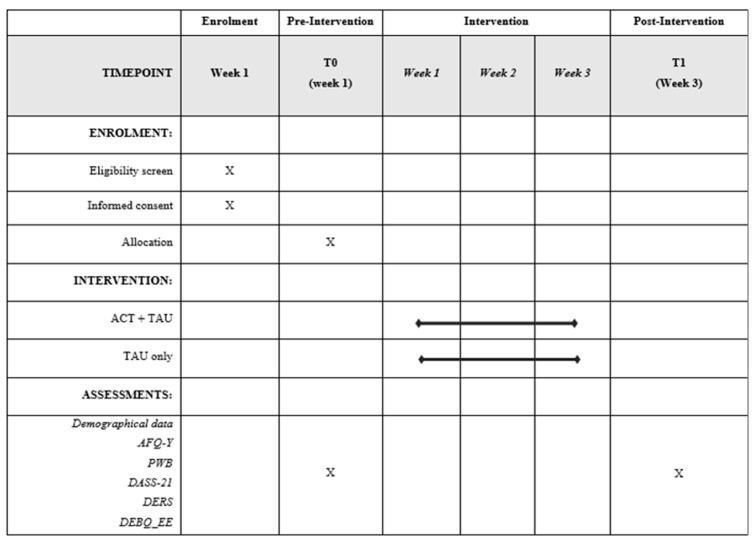
Spirit figure.

The schedule of enrolment, intervention, and assessment in the study is presented in Figure 1 (see also Supplementary Materials). 

### 2.6. The ACT-Based Intervention

Participants assigned to the ACT group will attend treatment as usual plus a brief ACT-based intervention comprising three sessions, provided once a week, lasting about one hour each. The proposed intervention was designed by the authors of the study. It was developed following the main ACT-based manuals with adjustments according to the users and the context of the study implementation [54,55,56]. The ACT-based intervention is aimed to promote acceptance, awareness, and engagement in valued behaviors, as alternatives to deal with dysfunctional thoughts and feelings. Experiential exercises and key metaphors will be used to target the core processes of the intervention. 

Specifically, we developed a practical and interactive intervention, which comprises practical activities such as roleplay and writing activities and imaginative activities supported by metaphors. The sessions are based on the use of age-appropriate language to convey the most complex therapeutic processes easily. According to the model of Strosahl and colleagues [57], sessions will respectively target the components of openness, awareness, and engagement. In the Openness session, young patients are guided to recognize distressing thoughts, emotions, or body sensations they are dealing with and imagine what their life would be like without their source of suffering. Then, they are invited to recognize their ineffective strategies they use for controlling or avoid pain instead of working on growing life. In the Awareness section, young patients are guided to learn how to feel in contact with the present internal states at the moment they are and just note what they are experiencing, without reacting. They are also invited to consider the self as a viewpoint, or a psychological space from which observing their thoughts and feelings without being overwhelmed. Finally, in the Engagement session, young patients are asked to list the personal qualities they have or those they desire to have for themselves. They are also guided to clarify their values, as chosen life direction they desire to pursue to become the person they want to be. Then, they are required to identify which behaviors need to be engaged in order to achieve their values.

A detailed description of the sessions is provided in Table 1.

### 2.7. Treatment Fidelity 

The research group comprises licensed psychologists, researchers, and doctoral students with expertise in clinical interventions in health care settings and research. 

Sessions will be administered by a licensed clinical psychologist with about three years of expertise in ACT clinical practice for adolescents both in individual and group settings blinded to research aims. 

The structure and the content of the sessions are consistent with ACT theory, as mentioned above. 

To ensure that the intervention will be delivered as planned, direct observation of the intervention is guaranteed. In line with previous studies [59] two independent bachelor-level observers blinded to research aims will observe at least 20% of sessions to evaluate their adherence to the protocol, after a period of training. By using a checklist, they will assess the coverage of the intervention’s components. Checklists include all the contents and the experiential exercises planned for each session. Coders have to achieve a minimum of 80% reliability with the expert trainers and each other. With a lower level of agreement, the data will be dismissed.

### 2.8. Sample Size Calculation

In line with previous studies [60,61] to calculate the sample size, an a priori power analysis was carried out using the software G.Power 3.1.9.4 [62]. 

The required sample size was computed for an analysis of variance (ANOVA) with 2 groups (between variable; ACT + TAU vs. TAU) × 2 times (within variable; Time 0/Time 1). To the best of the author’s knowledge, no other similar studies are available in the literature. Therefore, the a priori effect size (Cohen’s *f*) for the global effect (groups x time) was set to 0.2 (small effect size) [63]. Moreover, the Type I error [α] probability was set to 0.05, and the power [1-β] was set to 0.95. In addition, correlation among repeated measures to 0.3 (small–moderate correlation/linear effect). Non-sphericity was assumed, and it was set to 1. Results showed that there is a more than 95% chance of correctly rejecting the null hypothesis of no significant effect of the interaction with an overall sample of 116 subjects: a minimum of 58 participants per group.

### 2.9. Statistical Analysis 

Descriptive statistical analyses will be conducted to investigate the baseline characteristics of the sample. To evaluate differences between the two groups (ACT + TAU vs. TAU only) across the two time points (pre—Time 0/week 1; post—Time 1/week 3 psychological intervention), repeated measure between groups ANOVAs will be performed for each dependent variable—namely, AFQ-Y, PWB, DASS-21, DERS, and DEBQ. In this case, a global effect size (Cohen’s f and/or eta-squared—η^2^) will be used to quantify the [global] difference of the two groups across times. Global effects size will be interpreted with the following benchmarks [63]: null (*f* < 0.10; η^2^ < 0.003); small (f from 0.10 to 0.25; η^2^ from 0.003 to 0.039); moderate (*f* from 0.25 to 0.40; η^2^ from 0.40 to 0.110); and large (*f* ≥ 0.40; η^2^ > 0.110). Moreover, several focused comparisons will be performed to assess differences both within and between groups. In particular, differences between the two groups will be assessed using an independent samples t-test both at the baseline as well as at the end of psychological treatment, separately—namely, T1: ACT + TAU vs. TAU and T2: ACT + TAU vs. TAU. Conversely, differences within each group across the two time points will be assessed using dependent samples t-test—namely, ACT + TAU: T1 vs. T2; and TAU: T1 vs. T2. The magnitude of the differences will be interpreted by using the Cohen’s d [64]—more in detail, for between-groups comparisons, the classical Cohen’s d formula will be used; in contrast, for within-groups comparisons, the modified formula for dependent data will be used. Each Cohen’s d will be interpreted using the following benchmarks: null (*d* < 0.20), small (d from 0.20 to 0.49), moderate (d from 0.50 to 0.79), and strong (*d* > 0.80). 

Participants who reported missing data as well as subjects who will not participate in each therapy session will be excluded from the final sample, and thus, their data will not be analyzed. Moreover, also, participants who drop out from the program will be excluded from the final sample. 

Analyses will be carried out using Jamovi (version 1.6.15) [64].

## 3. Discussion

This work is aimed at describing the study that will be implemented to evaluate the efficacy of a brief ACT-based intervention in improving psychological conditions of youth with obesity within a context of a multidisciplinary in-hospital obesity rehabilitation programs. 

The dramatic global increase of childhood and adolescence obesity calls for a better understanding of the associated risk factors and requires effective treatments. Multidisciplinary interventions targeting healthy diet and physical activity are needed to treat obesity effectively [65]. However, since obesity impairs both the physical and psychological health of children and adolescents, one of the key focuses of the interventions should be psychological well-being and mental health. 

The majority of treatments are substantially conceived to prevent and control the widespread increase of childhood and adolescence obesity through parental education on proper nutritional requirements for their children and the implementation of healthy eating habits and physical activity [66]. Conversely, few studies have been implemented to provide psychological interventions, targeting emotional regulation in children and adolescents, which is essential for healthy psychological functioning [67] and healthy eating habits. In this regard, the present study has been conceived. 

The psychological intervention proposed in the current study will be implemented within a multidisciplinary in-hospital program for weight loss and obesity rehabilitation. Even if the high specificity of the context could represent a limitation for the generalizability of potential significant results, an inpatient rehabilitative program allows young people to live in an environment where they may eat healthily and engage in physical activity. This could be the setting of choice to improve psychological well-being and important self-regulation skills related to healthy habits in adolescents with obesity, according to the ACT framework.

Consistent results of this study would be able to open promising directions for future research. Specifically, by incorporating measurement of weight loss and planning a follow-up over time after the rehabilitation program, it will be possible to assess whether the intervention could be helpful to sustain long-term weight loss and long-term adoption of a healthy lifestyle.

## 4. Conclusions

Significant results from this study would provide additional information concerning how ACT can enhance the psychological conditions of adolescents with obesity, seeking treatment for weight loss and rehabilitation. 

## Figures and Tables

**Table 1:** The ACT-based intervention.

Session Number	Domains	Goals and Therapeutic Processes	Experiential Activities and Metaphors
Session 1 Week 1	Openness	The purpose of this session is to develop the willingness to experiencing distress and undesirable private events as part of human experience, without judgment or attempts to avoid or control internal states, even if they are unpleasant.	Experiential exercise: “How would my life be if…” In this experiential activity, young patients are guided to answer the key question: “How would my life be if I didn’t have … [my problem that I have]”. The problem could be their weight or anything else related to their condition of obesity or any other perceived problem in their life. Participants are encouraged to describe what they desire to do if they did not have obesity, in order to take distance, observe and accept their conditions and related thoughts and feelings.
Session 2 Week 2	Awareness	The purpose of this session is to promote the ability to be present at the moment and face events as contextually situated. To be aware means stepping back from suffering situations and seeing them in the context where they occur.	Metaphor: “The sky and weather metaphor” [58]. In this metaphor, personal thoughts and feelings are presented as the weather and the self as the sky. The weather naturally changes. Despite that, it can never harm or change the sky. No matter how bad the weather, the sky always has room for it. Sometimes, we forget that the sky is there, but it is still there. In the same way, difficult thoughts and feelings can occur. No matter how harmful they are, the self is still there. We can always learn how to access this part of us. It is a safe space that contains difficult thought and feelings.
Session 3 Week 3	Engagement	The purpose of this session is to foster values clarification and engagement in actions linked to personal values, such as relationships and personal growth. If a person engages himself in committed actions driven by chosen life directions, they can pursue a meaningful and coherent life.	Experiential exercise: “The treasure hunt”. In this experiential exercise, young patients are asked to draw a treasure hunt in which the treasure is a value in their life and the route is made of behaviors that need to be engaged to reach a meaningful life.

## Data Availability

The collected in this study will be available on request from author A.G.U. with the permission of author A.S. The data will not be publicly available due to privacy/ethical restrictions.

## Supplementary Materials

The following are available online at https://www.mdpi.com/article/10.3390/ijerph18126225/s1, Table S1: Spirit Checklist.
