# Supplementary material for: The ACTyourCHANGE in Teens Study Protocol: An Acceptance and Commitment Therapy-Based Intervention for Adolescents with Obesity: A Randomized Controlled Trial
Source: Int J Environ Res Public Health. 2021 Jun 9;18(12):6225. doi: 10.3390/ijerph18126225 (PMC8296029; doi:10.3390/ijerph18126225)
Supplement: Supplementary file 1 [file ijerph-18-06225-s001.zip › ijerph-1209000-SI.pdf]

SPIRIT

STANDARD PROTOCOL ITEMS: RECOMMENDATIONS FOR INTERVENTIONAL TRIALS

**Table S1.** SPIRIT 2013 Checklist: Recommended items to address in a clinical trial protocol and related documents*.

| Section/item | ItemNo | Description |
|---|---|---|
| | | **Administrative information** |
| Title | 1 | Descriptive title identifying the study design, population, interventions, and, if applicable, trial acronym<br>**The ACTyourCHANGE Study Protocol: an ACT-based intervention for adolescents with obesity. A Randomized Controlled Trial**<br>Page 1 |
| Trial registration | 2a | Clinical Trial Registration number: NCT04474509<br>Page 2 |
| | 2b | All items from the World Health Organization Trial Registration Data Set |
| Protocol version | 3 | ACTyourCHANGE Protocol version 1.0 Date: 29/03/2021<br>Page 3 |
| Funding | 4 | Sources and types of financial, material, and other support<br>Not applicable. The trial is self-funded |

| | | |
|---|---|---|
| | | Names, affiliations, and roles of protocol contributors<br>Anna Guerrini Usubini [a,b*], Roberto Cattivelli [a,b], Vanessa Bertuzzi [b], Giorgia Varallo [a,b], Alessandro Rossi [c,d], Clarissa Volpi [a], Michela Bottacchi [a], Sofia Tamini [e], Alessandra De Col [e], Giada Pietrabissa [a,b], Strefania Mannarini [c,d] , Gianluca Castelnuovo [a,b], Enrico Molinari [a,b], Alessandro Sartorio [e,f]<br><br>[a] *Istituto Auxologico Italiano IRCCS, Psychology Research Laboratory, Milan, Italy;*<br>[b]*Department of Psychology, Catholic University of Milan, Milan, Italy;*<br>[c] *Department of Philosophy, Sociology, Education, and Applied Psychology, Section of Applied Psychology, University of Padova, Padova, Italy*<br>[d] *Interdepartmental Center for Family Research, University of Padova, Padova, Italy*<br>[e] *Istituto Auxologico Italiano, IRCCS, Experimental Laboratory for Auxo-Endocrinological Research, Milan and Piancavallo (VB), Italy*<br>[f] *Istituto Auxologico Italiano, IRCCS, Division of Auxology, Piancavallo (VB), Italy*<br><br>Page 1 |
| Roles and responsibilities | 5a | AGU and RC conceived the study, planned the experimental design, and made a substantial contribution to the manuscript drafting; VB, and GV contributed to the manuscript drafting; CV, MB, ST, ADC CV, MB, ST, ADC will contribute to the implementation of the study, including the enrollment of patients; GP and AR helped to define the study design with a substantial contribution in statistical analysis and manuscript drafting; SM, GC, EM, and AS revised the manuscript. All authors read and approved the final manuscript.<br><br>Page 16 |

| | | |
|---|---|---|
| 5b | Name and contact information for the trial sponsor | Not applicable. There is no trial sponsor |
| 5c | Role of study sponsor and funders, if any, in study design; collection, management, analysis, and interpretation of data; writing of the report; and the decision to submit the report for publication, including whether they will have ultimate authority over any of these activities | Not applicable. There are no sponsors and funders |
| 5d | Composition, roles, and responsibilities of the coordinating centre, steering committee, endpoint adjudication committee, data management team, and other individuals or groups overseeing the trial, if applicable (see Item 21a for data monitoring committee) | Not applicable. Data monitoring committee is not needed and not common in psychology research. The Medical Ethic Committee of Istituto Auxologico Italiano approved the study including all the related procedures (also for data collection). Pag 11 |

**Introduction**

| | | |
|---|---|---|
| Background and rationale | 6a | Description of research question and justification for undertaking the trial, including summary of relevant studies (published and unpublished) examining benefits and harms for each intervention<br><br>Childhood obesity is a major public health concern. The complexity of obesity requires a broad multidisciplinary approach – composed of medical, nutritional, physical, and psychological components – to address adherence to a healthy lifestyle and improving psychological well-being, quality of life, and treating obesity-related comorbidities.<br>Acceptance and Commitment Therapy is a third-wave CBTs raised in the last twenty years. ACT aims to promote psychological flexibility, that is the ability to live in contact with the present moment and in a conscious way while implementing behaviors to live following one's values. Evidence from the literature shows that enhancing acceptance, openness, awareness, in other words psychological flexibility, is a key factor in promoting stable and lasting behavioral change, particularly in the development of healthy behaviors and improve self-regulation. Several ACT-based protocols have been applied to adolescents, with promising results. Unfortunately, there are not many studies in the literature concerning the application of an ACT protocol for adolescents with overweight/obesity problems.<br><br>Page 3-6 |

| | | |
|---|---|---|
| | 6b | Explanation for choice of comparators<br><br>A superiority Randomized Controlled Trial (RCT) with parallel groups will be conducted with an ACT-based intervention plus TAU compared with TAU only for adolescents with obesity attending a multidisciplinary rehabilitation program for weight loss.<br><br>Page 7 |
| Objectives | 7 | Specific objectives or hypotheses<br>The present study aims to evaluate the effectiveness of a brief ACT-based-intervention to improve psychological conditions in a sample of adolescents with obesity attending a multidisciplinary in-hospital rehabilitation program for weight loss.<br><br>Page 6 |

| | | |
|---|---|---|
| Trial design | 8 | Description of trial design including type of trial (eg, parallel group, crossover, factorial, single group), allocation ratio, and framework (eg, superiority, equivalence, noninferiority, exploratory)<br><br>A superiority Randomized Controlled Trial (RCT) with parallel groups will be conducted with an ACT-based intervention plus TAU compared with TAU only for adolescents with obesity attending a multidisciplinary rehabilitation program for weight loss. Participants will be randomly assigned to the experimental (ACT+ TAU) or control (TAU) group. We will perform a simple randomization with 1:1 allocation ratio using the Web site Randomization.com (http://www.randomization.com). Randomization will occur after the baseline measurements. Allocation concealment will be ensured by assigning since all the patients will generate an anonymous code that will be associated to the randomization generated by the program. Researchers will be blind to the association made.<br><br>Page 7, 9 |
| **Methods: Participants, interventions, and outcomes** | | |
| Study setting | 9 | Description of study settings (eg, community clinic, academic hospital) and list of countries where data will be collected. Reference to where list of study sites can be obtained<br><br>The study will be conducted in the context of a multidisciplinary rehabilitation program for weight loss in Istituto Auxologico Italiano IRCCS, Piancavallo (VB) a clinical center for the rehabilitation of obesity and related diseases, situated in the North of Italy.<br><br>Page 6 |

| | | |
|---|---|---|
| Eligibility criteria | 10 | Inclusion and exclusion criteria for participants. If applicable, eligibility criteria for study centres and individuals who will perform the interventions (eg, surgeons, psychotherapists)<br><br>Participants will be obese patients enrolled for a weight loss rehabilitation program in a clinical center in the North of Italy.  Patients of both genders will be included if they will meet these following inclusion criteria: 1) age between 12 and 17 years; 2) BMI>97$^{th}$ percentile; 3) Italian mother tongue; 4) written and informed consent to participate from both parents and written assent from participants.<br>Exclusion criteria for the study are: 1) other psychiatric disturbances (diagnosed according to DSM 5 criteria); 2) other medical conditions not related to obesity that could compromise participation at the study.<br><br>Page 7 |

| | | |
|---|---|---|
| Interventions | 11a | Interventions for each group with sufficient detail to allow replication, including how and when they will be administered<br><br>The trial will be conducted within the context of a three-week in-hospital multidisciplinary rehabilitation program for weight loss based on nutritional, physical, and psychological rehabilitation. As part of the program, adolescents will be assessed by a staff dietician and placed on an individualized hypocaloric balanced Mediterranean diet. They will follow a nutritional counselling aimed at promoting the adoption of a healthy lifestyle; Moreover, they will attend a physical activity program consisting of five training sessions per week lasting one hour each with trainers; In addition, they will be involved in psychological counselling provided once a week by a clinical psychologist, aimed to address psychological factors related to dysfunctional lifestyle habits. At week 1 of the hospitalization, all the patients will be screen for admission at the study; within an interview, a clinical psychologist member of research team will meet inpatient adolescents providing them information about the study and assessing the eligibility criteria. Once obtained informed consent to participate from parents and written assent from participants, young patients recruited for the study will be randomly assigned into two conditions. The experimental group will attend TAU plus a brief ACT-based intervention, that comprises three sessions, provided once a week, lasting about one hour each, while the control group will attend TAU only. At pre (Time 0/week 1) and post psychological intervention (Time 1/week 3), patients of both conditions, at the same time, in a room used for research in the hospital, are invited to fill the questionnaires, under the supervision of a member of research team.<br>In case of any form of psychological discomfort due to the participation to the intervention, or any doubt, or need for information concerning the trial, participants can consult the [illegible] |

| | | |
|---|---|---|
| | 11b | Criteria for discontinuing or modifying allocated interventions for a given trial participant (eg, drug dose change in response to harms, participant request, or improving/worsening disease)<br><br>We do not expect any adverse or unintended effects due to the trial participation, However, In case of any form of psychological discomfort due to the participation to the intervention, or any doubt, or need for information concerning the trial, participants can consult the psychologist responsible for the study. Once enrolled, patients may withdraw from the study at any time. This will not affect their future treatment.<br><br>Page 10-11 |

| | | |
|---|---|---|
| | 11c | Strategies to improve adherence to intervention protocols, and any procedures for monitoring adherence (eg, drug tablet return, laboratory tests)<br><br>The research group comprises licensed psychologists, researchers, and doctoral students with expertise in the field of clinical interventions in health care settings and research.<br>Sessions will be administered by a licensed clinical psychologist with about three years of expertise in ACT clinical practice for adolescents both in individual and group settings, blinded to research aims.<br>The structure and the content of the sessions are consistent with ACT theory.<br>In line with previous studies (Villatte et al., 2016), two bachelor-level observers, also blinded to research aims, will attend at least 20 % of sessions to evaluate the adherence to the protocol and frequency and depth of coverage of the intervention's components, after a period of training. They will use a checklist detailed for all the contents, the targeted intervention's components, and experiential exercises planned for each session. Coders have to achieve a minimum of 80% reliability with the expert trainers and each other. With a lower level of agreement, the data will be dismissed.<br><br>Page 12 |

| | | | |
|---|---|---|---|
| | 11d | Relevant concomitant care and interventions that are permitted or prohibited during the trial | As part of the program, during the inpatient phase adolescents of both groups, are assessed by a staff dietician and placed on an individualized hypocaloric balanced Mediterranean diet. They also follow a nutritional counselling aimed at promoting the adoption of a healthy lifestyle; Moreover, they attend a physical activity program consisting of five training sessions per week lasting one hour each with trainers; In addition, they are involved in psychological counselling provided once a week by a clinical psychologist, aimed to address psychological factors related to dysfunctional lifestyle<br><br>Page 10 |

Primary, secondary, and other outcomes, including the specific measurement variable (eg, systolic blood pressure), analysis metric (eg, change from baseline, final value, time to event), method of aggregation (eg, median, proportion), and time point for each outcome. Explanation of the clinical relevance of chosen efficacy and harm outcomes is strongly recommended

Primary psychological outcomes are PWB.
Secondary outcomes include AFQ-Y, DASS-21, DERS, DEBQ.

All the measures will be collected at pre (Time 0/week 1) and post psychological intervention (Time 1/week3).

Page 7-8

| | | |
|---|---|---|
| Participant timeline | 13 | Time schedule of enrolment, interventions (including any run-ins and washouts), assessments, and visits for participants. A schematic diagram is highly recommended (see Figure)<br><br>See SPIRIT figure |
| Sample size | 14 | Estimated number of participants needed to achieve study objectives and how it was determined, including clinical and statistical assumptions supporting any sample size calculations<br><br>In line with previous studies, to calculate the sample size, an a priori power analysis was carried out using the software G.Power 3.1.9.4 (Faul et al., 2007). To the author's knowledge, no other similar studies are available in the literature. Therefore, sample size has been calculated for an analysis of variance (ANOVA) with 2x2 repeated measures (variable between experimental group/control group; variable within Time0/Time1), setting $\alpha$ to 0.05, power (1- β) to 0.80 (Cohen, 1988), correlation among repeated measures to 0.5, and non-sphericity correction to 1, the total number of subjects required to obtain a small effect size (f2 = 0.20) is 50.<br><br>Page 12 |

| | | |
|---|---|---|
| Recruitment | 15 | Strategies for achieving adequate participant enrolment to reach target sample size<br><br>Participants will be selected among the patients hospitalized at Istituto Auxologico Italiano, IRCCS; Piancavallo (VB) for a body weight reduction program. Consecutive in-patients will be screened for admission to the study while attending a three-week weight-loss rehabilitation program.<br><br>Page 7 |

**Methods: Assignment of interventions (for controlled trials)**

| | | |
|---|---|---|
| Allocation: | | |
| Sequence generation | 16a | Method of generating the allocation sequence (eg, computer-generated random numbers), and list of any factors for stratification. To reduce predictability of a random sequence, details of any planned restriction (eg, blocking) should be provided in a separate document that is unavailable to those who enrol participants or assign interventions<br><br>Participants will be randomly assigned to the experimental (ACT+ TAU) or control (TAU) group. We will perform a simple randomization with 1:1 allocation ratio using the Web site Randomization.com (http://www.randomization.com). Randomization will occur after the baseline measurements.<br><br>Page 9 |

| | | |
|---|---|---|
| Allocation concealment mechanism | 16b | Mechanism of implementing the allocation sequence (eg, central telephone; sequentially numbered, opaque, sealed envelopes), describing any steps to conceal the sequence until interventions are assigned<br><br>Allocation concealment will be ensured since all the patients will generate an anonymous code that will be associated to the randomization's sequence generated by the program. Researchers will be blind to the association made.<br><br>Page 9 |
| Implementation | 16c | Who will generate the allocation sequence, who will enrol participants, and who will assign participants to interventions<br><br>CV, MB, ST, ADC, will contribute to the implementation of the study, including the enrollment of patients.<br><br>Page 16 |
| Blinding (masking) | 17a | Who will be blinded after assignment to interventions (eg, trial participants, care providers, outcome assessors, data analysts), and how<br><br>The clinical psychologist who will conduct the sessions, the participants and the observers will be blind to research aims.<br><br>Page 11 |
| | 18b | If blinded, circumstances under which unblinding is permissible, and procedure for revealing a participant's allocated intervention during the trial<br><br>Not applicable. Unblinding is not permissible |

**Methods: Data collection, management, and analysis**

| | | |
|---|---|---|
| Data collection methods | 18a | Plans for assessment and collection of outcome, baseline, and other trial data, including any related processes to promote data quality (eg, duplicate measurements, training of assessors) and a description of study instruments (eg, questionnaires, laboratory tests) along with their reliability and validity, if known. Reference to where data collection forms can be found, if not in the protocol<br><br>All demographical (gender, age) and clinical variables will be collected via self-report form. Clinical variables will be collected as follows:<br>Psychological conditions:<br>- Psychological Well-Being. The *Psychological Well-Being Scales* (PWB)<br>- Experiential avoidance and fusion. The *Avoidance and Fusion Questionnaire for Youth* (AFQ-Y)<br>- Psychological distress. The *Depression Anxiety Stress Scale* (DASS-21)<br>- Emotional regulation. The *Difficulties in Emotion Regulation Scale* (DERS)<br>- Emotional eating. The *Emotional Eating subscale* of the *Dutch Eating Behavior Questionnaire* (DEBQ)<br><br>Page 7-9 |
| | 18b | Plans to promote participant retention and complete follow-up, including list of any outcome data to be collected for participants who discontinue or deviate from intervention protocols<br><br>Not applicable. We do not plan any procedure to promote participant retention |

| | | |
|---|---|---|
| | | Plans for data entry, coding, security, and storage, including any related processes to promote data quality (eg, double data entry; range checks for data values). Reference to where details of data management procedures can be found, if not in the protocol |
| Data management | 19 | Completed questionnaires will be stored in a room used for research in the hospital which is accessible only for research team members. Data will be stored on password-protected files kept for five years after the end of the trial. |
| | | Page 11 |

| | | |
|---|---|---|
| Statistical methods | 20a | Statistical methods for analysing primary and secondary outcomes. Reference to where other details of the statistical analysis plan can be found, if not in the protocol<br><br>Data will be analyzed basing on intention – to – treat (ITT) method. ITT is a method for analyzing results in which all randomized participants are included in the statistical analysis according to the groups to which they are randomly assigned, regardless of the treatment they received, or the subsequent withdrawal from treatment or any deviation from the protocol (McCoy, 2017).<br>As for missing data, to assess if missing data will follow a Missing Completely At Random (MCAR) mechanism, Little's MCAR test will be performed. Missing data lower than 5% will be considered negligible. In case of further amount of missing data, multiple imputations will be used. Descriptive statistical analyses will be conducted to investigate the baseline characteristics of the sample. To evaluate differences between the two groups (ACT+TAU vs. TAU only)in the variables of interest (AFQ-Y, PWB, DASS-21, DERS, DEBQ) at pre (Time 0)/week1) and post (Time 1(week 3) psychological intervention, mixed between within analysis of variances (ANOVAs) (2x2) will be conducted. Changes in mean scores of AFQ, PWB, DASS-21, DERS, and DEBQ, from Time 0 to Time 1 will be assessed. As for effect size, Cohen's *d* will be calculated. Analyses will be carried out using Jamovi (version 1.2).<br><br>Page 13 |
| | 20b | Methods for any additional analyses (eg, subgroup and adjusted analyses)<br><br>Not applicable. We do not plan any additional analyses |

| | | |
|---|---|---|
| | 20c | Definition of analysis population relating to protocol non-adherence (eg, as randomised analysis), and any statistical methods to handle missing data (eg, multiple imputation)<br><br>Data will be analyzed basing on intention – to – treat. This analysis includes all randomized patients in the groups to which they are randomly assigned, regardless of their adherence with the entry criteria, regardless of the treatment they actually received, and regardless of subsequent withdrawal from treatment or deviation from the protocol. To assess if missing data will follow a Missing Completely At Random Mechanism, Little's MCAR test will be performed. Missing data lower than 5% will be considered negligible.<br><br>Page 13 |
| | **Methods: Monitoring** | |
| Data monitoring | 21a | Composition of data monitoring committee (DMC); summary of its role and reporting structure; statement of whether it is independent from the sponsor and competing interests; and reference to where further details about its charter can be found, if not in the protocol. Alternatively, an explanation of why a DMC is not needed<br><br>Not applicable. The Medical Ethics Committee of Istituto Auxologico Italiano approved the study (registration number 2021_01_26_03). The study personnel and the Ethical Committee will ensure that the study is conducted within appropriate professional, ethical guidelines, ensuring that Good Clinical Practice guidelines are observed<br><br>Page 15 |

| | | |
|---|---|---|
| | 21b | Description of any interim analyses and stopping guidelines, including who will have access to these interim results and make the final decision to terminate the trial<br><br>Not applicable. We do not plan any interim analyses |
| Harms | 22 | Plans for collecting, assessing, reporting, and managing solicited and spontaneously reported adverse events and other unintended effects of trial interventions or trial conduct<br><br>We do not expect any adverse or unintended effects due to the trial participation, However, in case of any form of psychological discomfort due to the participation to the intervention, or any doubt, or need for information concerning the trial, participants can consult the psychologist responsible for the study. Once enrolled, patients may withdraw from the study at any time. This will not affect their future treatment<br><br>Page 10-11 |
| Auditing | 23 | Frequency and procedures for auditing trial conduct, if any, and whether the process will be independent from investigators and the sponsor<br><br>Not applicable. We do not plan any auditing trial conduct |
| | **Ethics and dissemination** | |
| Research ethics approval | 24 | Plans for seeking research ethics committee/institutional review board (REC/IRB) approval<br><br>The Medical Ethics Committee of Istituto Auxologico Italiano approved the study (registration number 2021_01_26_03).<br><br>Page 15 |

| | | |
|---|---|---|
| Protocol amendments | 25 | Plans for communicating important protocol modifications (eg, changes to eligibility criteria, outcomes, analyses) to relevant parties (eg, investigators, REC/IRBs, trial participants, trial registries, journals, regulators)<br><br>Not applicable. We do not plan important protocol modifications |
| Consent or assent | 26a | Who will obtain informed consent or assent from potential trial participants or authorised surrogates, and how (see Item 32)<br><br>At week 1 of the hospitalization, all the patients will be screen for admission at the study; within an interview, a clinical psychologist member of research team will meet inpatient adolescents providing them information about the study and assessing the eligibility criteria. Once obtained informed consent to participate from parents and written assent from participants, young patients recruited for the study will be randomly assigned into experimental conditions.<br><br>Page 10 |
| | 26b | Additional consent provisions for collection and use of participant data and biological specimens in ancillary studies, if applicable<br><br>Not applicable. No biological specimens will be collected as part of this trial. |
| Confidentiality | 27 | How personal information about potential and enrolled participants will be collected, shared, and maintained in order to protect confidentiality before, during, and after the trial<br><br>Completed questionnaires will be stored in a room used for research in the hospital which is accessible only for research team members. Data will be stored on password-protected files kept for five years after the end of the trial.<br><br>Page 11 |

| | | |
|---|---|---|
| Declaration of interests | 28 | Financial and other competing interests for principal investigators for the overall trial and each study site<br><br>The authors declare that they have no competing interests<br><br>Page 15 |
| Access to data | 29 | Statement of who will have access to the final trial dataset, and disclosure of contractual agreements that limit such access for investigators<br><br>Only researchers involved into the study will have access to the final trial dataset<br><br>Page 15 |
| Ancillary and post-trial care | 30 | Provisions, if any, for ancillary and post-trial care, and for compensation to those who suffer harm from trial participation<br>We do not expect any adverse or unintended effects due to the trial participation, However, in case of any form of psychological discomfort due to the participation to the intervention, or any doubt, or need for information concerning the trial, participants can consult the psychologist responsible for the study. Once enrolled, patients may withdraw from the study at any time. This will not affect their future treatment<br><br>Page 10-11 |

| | | |
|---|---|---|
| Dissemination policy | 31a | Plans for investigators and sponsor to communicate trial results to participants, healthcare professionals, the public, and other relevant groups (eg, via publication, reporting in results databases, or other data sharing arrangements), including any publication restrictions<br><br>We plan to communicate trial results to participants with a final report provided via e-mail. Results will be available for the scientific communication and health care experts via publication in scientific journals and participation in national and international congresses. We do not plan to deliver a completely de-identified dataset.<br>plan to communicate trial results to participant with a final report provided via e-mail.<br><br>Page 15 |
| | 31b | Authorship eligibility guidelines and any intended use of professional writers<br><br>Not applicable. We do not use professional writers. All the researchers involved in the study are included in the authors list. All the authors participated in the trial implementation and authors' contribution is described. |
| | 31c | Not applicable. This research did not receive any specific grant from funding agencies in the public, commercial, or not-for-profit sectors. |
| **Appendices** | | |
| Informed consent materials | 32 | Model consent form and other related documentation given to participants and authorised surrogates<br><br>Informed consent will be obtained by parents of participants. |

| | | |
|---|---|---|
| Biological specimens | 33 | Plans for collection, laboratory evaluation, and storage of biological specimens for genetic or molecular analysis in the current trial and for future use in ancillary studies, if applicable<br><br>Not applicable. No biological specimens will be collected |

*It is strongly recommended that this checklist be read in conjunction with the SPIRIT 2013 Explanation & Elaboration for important clarification on the items. Amendments to the protocol should be tracked and dated. The SPIRIT checklist is copyrighted by the SPIRIT Group under the Creative Commons "Attribution-NonCommercial-NoDerivs 3.0 Unported" license.
